# Chili pepper aspiration in elderly patients: a case series highlighting diagnostic challenges and the role of cryoextraction

**DOI:** 10.3389/fmed.2026.1751021

**Published:** 2026-02-18

**Authors:** Qun-cuo Zeren, Bijun Yang, Yang Bai

**Affiliations:** 1 Department of Respiratory Medicine, Changdu People’s Hospital of Xizang, Changdu, Tibet Autonomous Region, China; 2 Department of Respiratory and Critical Care Medicine, The First Affiliated Hospital of Chongqing Medical University, Chongqing, China

**Keywords:** chili pepper, foreign body aspiration, interventional pulmonology, postgraduate education, transbronchial cryoextraction

## Abstract

**Background:**

Foreign body aspiration is a recognized clinical emergency, but chili pepper fragment inhalation remains an under-recognized etiology, particularly in elderly populations. This study aims to clarify the clinical and radiological characteristics, diagnostic challenges, and bronchoscopic management of such cases.

**Methods:**

We report two cases of elderly patients with subacute respiratory symptoms initially indicative of obstructive pneumonia or neoplasia. Both underwent extensive evaluation, including serial computed tomography (CT) and bronchoscopy.

**Results:**

In both cases, high-resolution CT scans demonstrated hyperdense, V-shaped, or annular opacities within bronchial structures (best visualized with mediastinal window settings) that were suggestive of an inhaled foreign body. Prominent indirect indicators included localized bronchial wall thickening, luminal narrowing, and post-obstructive parenchymal changes. Flexible bronchoscopy identified chili pepper fragments obstructing the distal airways. Cryoextraction successfully removed specimens intact without fragmentation. Follow-up revealed residual bronchiectasis but significant symptomatic improvement.

**Conclusion:**

Chili pepper fragment inhalation may be considered in cases of unexplained pneumonia, particularly in elderly populations or those with dietary habits involving small, sharp food items. CT imaging of hyperdense, V-shaped, or annular opacities within bronchial structures might provide valuable diagnostic clues, though radiographic findings may be subtle and non-pathognomonic. Bronchoscopy remains a key diagnostic and therapeutic modality, with cryoextraction demonstrating efficacy for extraction. Increased clinical awareness and prompt intervention are essential to prevent complications.

## Introduction

In respiratory medicine, foreign body aspiration remains a significant clinical concern, particularly affecting vulnerable populations such as the elderly and children (1, 2). Among aspirated objects, organic materials like chili pepper fragments are less frequent but clinically impactful, posing unique diagnostic and management challenges due to their insidious and nonspecific manifestations (3). Patients with chili pepper aspiration may present with chronic cough, recurrent pneumonia, or lobar collapse—symptoms that overlap with common respiratory conditions (e.g., asthma, bronchitis, malignancy), leading to delayed or missed diagnoses (4). This diagnostic ambiguity is particularly exacerbated by the absence of a clear aspiration history in many cases, especially among elderly patients with age-related dysphagia or neurological comorbidities (5). The delayed diagnosis of chili pepper aspiration might result in severe respiratory complications, including obstructive pneumonia, chronic inflammation, and bronchiectasis (6, 7).

Radiologically, chili pepper fragments may exhibit hyperdense V-shaped, U-shaped, or annular opacities within the bronchial lumen, best visualized via mediastinal window computed tomography (CT) settings (7). These features are non-pathognomonic but may aid in narrowing the differential diagnosis. But their distal migration and associated inflammatory responses—such as granulation tissue formation, mucosal edema, and purulent secretions—can render radiological detection and obscure bronchoscopic visualization (4). Bronchoscopy remains the preferred method for diagnosis and management, with cryoextraction being highly effective in retrieving fragile organic fragments intact while minimizing residual inflammation (8, 9).

Despite these insights, chili pepper aspiration is underreported and often overlooked in differential diagnoses. Current literature lacks comprehensive studies focusing on the radiological nuances, diagnostic challenges, and bronchoscopic management of such cases, particularly in adult populations. This gap contributes to delayed diagnosis and misclassification as common respiratory disorders. We present two cases to highlight key diagnostic and therapeutic insights, aiming to enhance clinical awareness.

## Methods

We report two cases of elderly patients with subacute respiratory symptoms initially indicative of obstructive pneumonia or neoplasia. Both patients had an extensive evaluation, including serial CT and bronchoscopy. This retrospective case series involves the analysis of existing clinical data from patients with chili pepper aspiration, without any experimental interventions. Ethical approval for this study was waived by the Institutional Review Boards of The First Affiliated Hospital of Chongqing Medical University due to its retrospective nature and lack of experimental procedures. Written informed consent for publication of clinical details and imaging data has been obtained from all patients. All identifying information has been anonymized to ensure patient confidentiality.

## Case presentation

### Case 1

**Figure 1:**
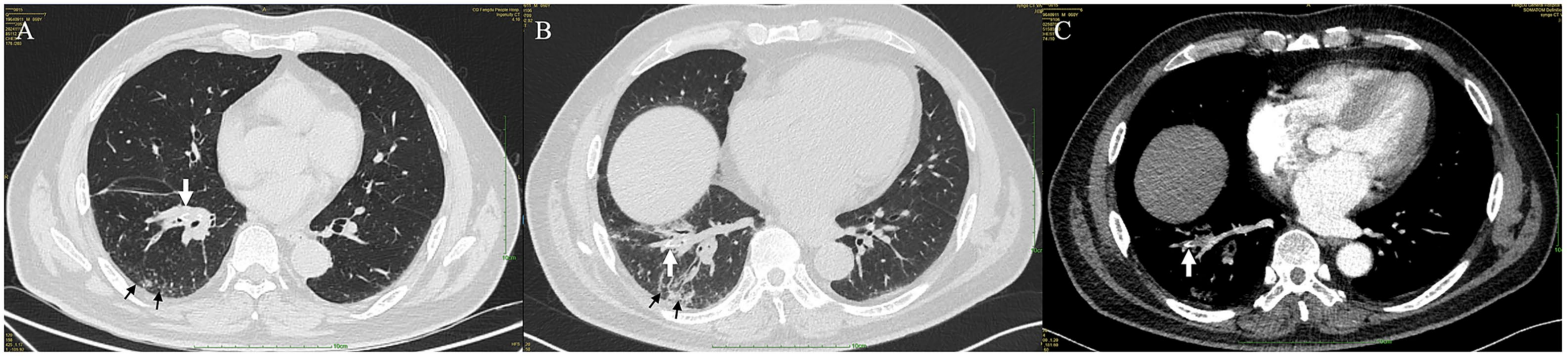
Serial chest computed tomography (CT) imaging of Case 1. **(A)** Initial axial CT scan (lung window) demonstrated circumferential wall thickening and narrowing of the right lower lobe bronchus (white arrow), with distal patchy ground-glass opacities (black arrow). Note the V-shaped hyperdense opacity (white arrowhead). **(B)** Follow-up axial CT scan (lung window) 8 months later revealed progression of bronchial wall thickening and luminal narrowing (white arrow), increased peripheral consolidation (black arrow), and distal migration of the V-shaped hyperdense opacity (white arrowhead). **(C)** Mediastinal-window setting of the follow-up CT scan more clearly visualized the V-shaped hyperdense opacity (white arrowhead), highly suggestive of an aspirated foreign body.

A 60-year-old man was referred to our department for 8 months of non-productive, paroxysmal cough (without hemoptysis, chest pain, fever, or dyspnea). Initial contrast-enhanced chest CT (lung window) at an outside hospital reported circumferential wall thickening, narrowing of the right lower lobe (RLL) bronchus (Figure 1A, white arrow), and distal patchy ground-glass opacities (Figure 1A, black arrow). Empiric moxifloxacin (400 mg daily for 14 days) targeting common pathogens of community-acquired pneumonia yielded modest symptomatic improvement; however, a repeat CT scan (lung window) 8 months later demonstrated progressive bronchial wall thickening, luminal narrowing (Figure 1B, white arrow), and increased peripheral consolidation (Figure 1B, black arrow), which raised concern for an underlying neoplasm and obstructive pneumonia. Flexible bronchoscopy at the local hospital identified an endobronchial mass occluding 80% of the orifice of the RLL anterior basal segment. A forceps biopsy confirmed chronic inflammation without evidence of malignancy, prompting transfer to our institution for further evaluation.

Upon admission, vital signs were within normal limits. Physical examination demonstrated reduced breath sounds over the RLL; no wheezes, rales, or rhonchi were auscultated. The laboratory tests were within normal limits. Retrospective review of the initial CT (lung window) uncovered a V-shaped hyperdense opacity at the distal margin of the thickened RLL anterior basal segment (Figure 1A, white arrowhead). Follow-up CT (lung window) showed distal migration of this opacity (Figure 1B, white arrowhead), which was more clearly visualized within the bronchus (Figure 1C, white arrowhead) on mediastinal-window settings. These radiologic findings were consistent with obstructive atelectasis secondary to a foreign body aspiration.

**Figure 2:**
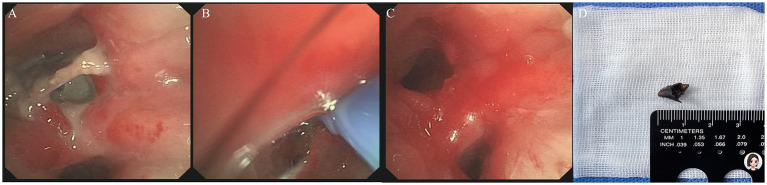
Bronchoscopic cryoextraction in Case 1. **(A)** Bronchoscopic view (3.7 mm bronchoscope) after granulation tissue debridement identified a chili pepper fragment lodged within the anterior basal airway of the right lower lobe. **(B)** A cryoprobe was advanced and inserted into the chili pepper lumen to facilitate freeze-adherence and cryoextraction. **(C)** Post-extraction bronchoscopic view confirmed complete removal of the foreign body, with no residual fragments. **(D)** The retrieved foreign body, measuring approximately 1 cm in length, was confirmed to be a fried chili pepper fragment.

A 5.9 mm bronchoscope (with a 2.8 mm working channel) was advanced to clear granulation tissue within the RLL anterior basal segment. A 3.7 mm bronchoscope (with a 2.0 mm working channel) was inserted into the subsegment. Saline instillation facilitated lumen dilation and visualization. A chili pepper fragment was identified within the RLL anterior basal segment (Figure 2A). Using a cryoprobe inserted into the fragment (Figure 2B), the intact foreign body was extracted via freeze-adherence. Post-procedural bronchoscopy confirmed no residual foreign body fragments in the distal RLL anterior basal segment (Figure 2C). The retrieved specimen was a 1 cm section of a fried chili pepper fragment (Figure 2D). The patient was discharged without further follow-up in our institution. The chest CT scan 1 month after treatment revealed bronchiectasis in the RLL with scattered patchy opacities. At 5 months of telephone follow-up, the patient was satisfied with the treatment and had no special discomfort.

### Case 2

**Figure 3:**
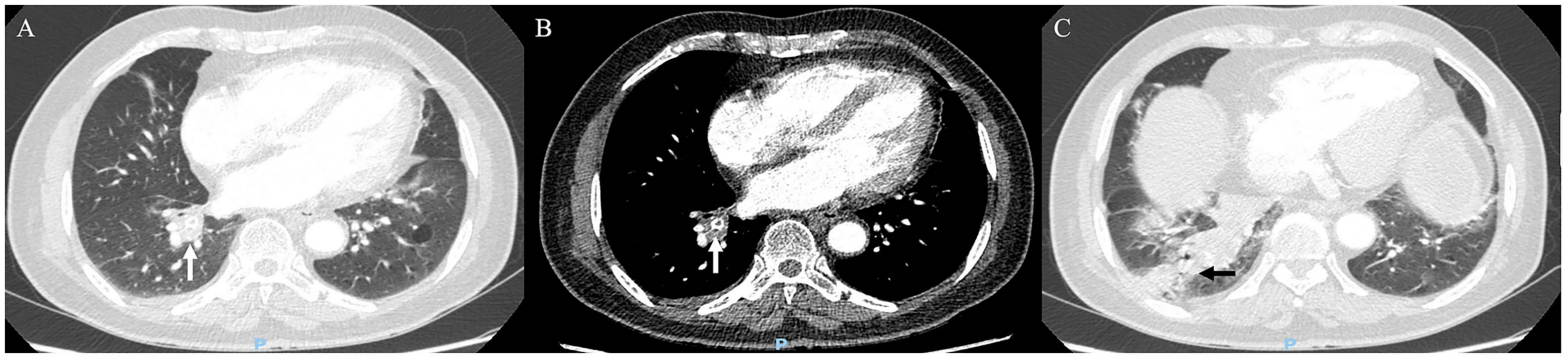
Serial chest computed tomography (CT) imaging of Case 2. **(A)** Axial CT scan (lung window) showed a hyperdense annular focus (white arrowhead) at the opening of the posterior basal segment of the right lower lobe. **(B)** Mediastinal-window setting accentuated the hyperdense annular focus (white arrowhead), strongly suggesting an aspirated foreign body. **(C)** Axial CT scan (lung window) showed associated post-obstructive atelectasis of the right lower lobe posterior basal segments, with dilated, mucus-filled bronchi (black arrow).

A 73-year-old man presented with 4 months of transient right lower chest pain and 1 week of productive paroxysmal cough (without hemoptysis, fever, or dyspnea). Contrast-enhanced chest CT (lung window) from an outside hospital revealed a hyperdense annular focus (Figure 3A, white arrowhead) at the opening of the RLL posterior basal segment, which was accentuated on mediastinal-window settings (Figure 3B, white arrowhead). The imaging findings (lung window) revealed concomitant atelectasis in the distal RLL, characterized by dilated bronchi containing mucus retention (Figure 3C, black arrow). This pattern strongly raised clinical concern for an inhaled foreign body.

**Figure 4:**
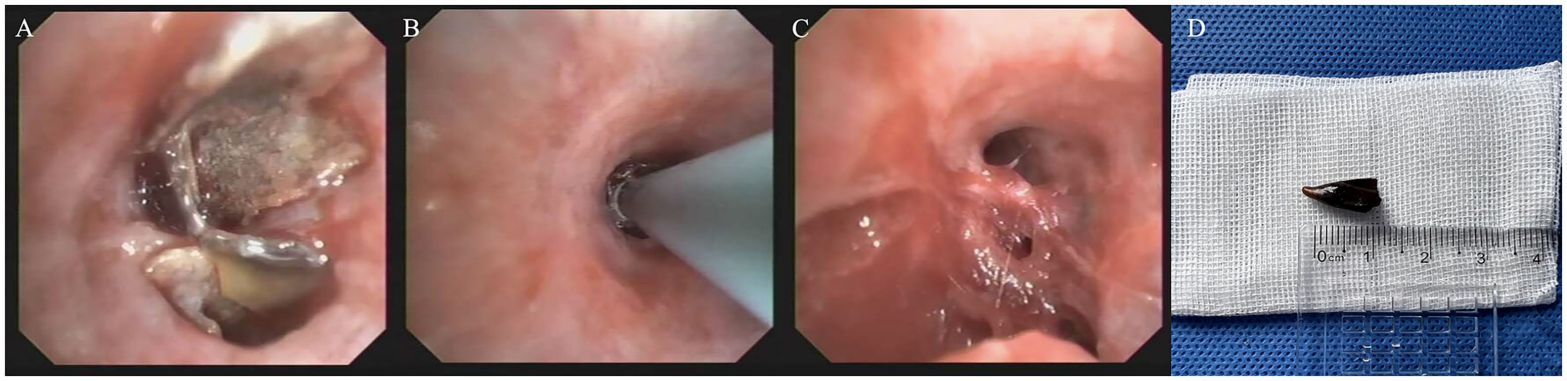
Bronchoscopic cryoextraction in Case 2. **(A)** Bronchoscopic view identified a chili pepper fragment lodged in the right lower lobe anterior basal airway. **(B)** A cryoprobe was advanced and inserted into the chili pepper fragment for cryoextraction. **(C)** Post-extraction bronchoscopic view confirmed complete removal of the foreign body, with no residual fragments. **(D)** The extracted foreign body, measuring approximately 1 cm, was identified as a fried chili pepper fragment.

Physical examination indicated diminished breath sounds over the RLL; no adventitious sounds were detected. Laboratory parameters were unremarkable. Bronchoscopy confirmed the presence of a chili pepper fragment within the RLL posterior basal segment (Figure 4A). Cryoextraction with a cryoprobe inserted into the chili pepper (Figure 4B) removed the intact fragment without fragmentation (Figure 4C). The retrieved specimen was a 1 cm section of a fried chili pepper fragment (Figure 4D). The patient was discharged without further follow-up in our institution. During the 3-month telephone follow-up, the patient expressed satisfaction with the treatment and reported no specific discomfort.

## Discussion

Chili pepper aspiration, though uncommon, represents a potentially serious respiratory event with distinct clinical and radiological features (6, 8). Clinically, patients often present with paroxysmal cough (productive or non-productive), which may be accompanied by chest pain, dyspnea, or symptoms of obstructive pneumonia (e.g., fever, purulent sputum), though these can be absent initially (10). A notable characteristic is the frequent lack of a clear recall of a choking episode, particularly among elderly individuals. This aspect is attributed to age-related decline in swallowing coordination, diminished cough/gag reflexes, underlying neurological conditions (e.g., post-stroke sequelae, dementia), and the use of sedating medications (5, 11–13). This, combined with symptom overlap with common respiratory disorders, often leads to misdiagnosis and delayed intervention (14). Consequently, a high index of suspicion is warranted for elderly patients with unexplained pneumonia, and a detailed dietary history (including ingestion of spicy foods) should be proactively sought (15). Preventive measures are paramount, such as advising against rapid eating or conversing while eating and recommending thorough chewing, especially for individuals with known swallowing difficulties (16).

The radiological presentation on chest CT is particularly noteworthy and might provide crucial diagnostic clues. Inhaled chili pepper fragments, often with a conical or tapered morphology linked to Chinese culinary preparation, frequently manifest as hyperdense V-shaped, U-shaped, or annular opacities within the bronchial lumen (7). This direct appearance, best visualized under mediastinal window settings, is suggestive but not specific to chili pepper fragment aspiration. Additional indirect CT signs may include localized bronchial wall thickening, luminal narrowing, peripheral consolidation, post-obstructive atelectasis, ground-glass opacities, bronchiectasis, and even fibrotic streaks distal to the obstruction site (4). However, compared to other inhaled foreign bodies, chili pepper fragments tend to migrate into distal bronchioles, rendering them potentially undetectable on CT imaging (7). Over time, prolonged retention can elicit a significant inflammatory response, leading to granulation tissue formation, mucosal edema, and purulent secretions that further obscure the foreign body. Notably, some organic foreign bodies may remain radiolucent even on multidetector CT (17). Thus, diagnostic bronchoscopy should be strongly considered in patients with clinical suspicion, even in the absence of direct radiological findings.

Beyond the physical obstruction of airways, capsaicin—the primary capsaicinoid in chili peppers—may play a critical role in exacerbating respiratory symptoms. As a well-established bronchial irritant, capsaicin activates transient receptor potential vanilloid-1 (TRPV1) receptors on airway mucosal nerves, triggering a cholinergic vagal reflex that promotes pro-inflammatory cytokine production and epithelial cell death (18). This direct stimulatory effect elicits classic asthma-like symptoms, including coughing, wheezing, and chest distress (19). These symptoms are clinically indistinguishable from allergic asthma episodes and lack specific diagnostic biomarkers for differential identification (20). However, patients in our study may not have experienced amplified airway irritation from capsaicin, owing to Chinese culinary practices. Frying chili peppers reduces their capsaicin content by approximately 39% to 53% via heat degradation and oil solubility, making them less pungent than their raw counterparts (21). This heat-induced decrease in capsaicin bioavailability likely mitigates the severity of TRPV1-mediated airway responses compared to aspiration of raw chili pepper fragments.

For respiratory physicians, familiarity with this CT appearance of chili pepper fragments (hyperdense, sharply defined intracorporeal opacities with distinct shapes) may facilitate prompt consideration of this diagnosis, though these findings are non-pathognomonic, as they may overlap with other small organic foreign bodies (e.g., nut fragments, plant debris). Definitive management relies on bronchoscopic extraction, a procedure that often requires meticulous clearance of proximal inflammatory granulation tissue and copious secretions to adequately visualize the embedded foreign body. In some cases, saline instillation through the bronchoscope’s working channel can help dilate the airway and improve visualization. A thinner, examination-grade bronchoscope may be necessary to navigate into subsegmental bronchi for detecting and removing distal objects (22).

Among bronchoscopic techniques, cryoextraction utilizing a cryoprobe is particularly well-suited for chili pepper fragment removal. This method relies on rapid ice crystal formation induced by Joule-Thomson expansion of cryogenic gases, which promotes probe adhesion to adjacent tissue and enables intact removal of water-rich foreign bodies (23–25). Chili pepper fragments, with rough surfaces that trap water or mucus, accelerate ice crystal growth and cryoadhesion, enhancing the efficacy of this technique. During the procedure, the cryoprobe is carefully inserted into the chili pepper’s lumen while avoiding contact with normal bronchial mucosa. This allows for intact extraction and minimizes mucosal injury and subsequent bleeding (9). While highly effective, cryoextraction depends on specialized equipment (e.g., cryoprobes, gas tanks) and operator expertise (23). In settings where a cryoprobe is unavailable, careful extraction using biopsy forceps is an alternative, but it mandates extreme gentleness to avoid fragmenting the brittle pepper, which could complicate the procedure and leave remnants behind (6). Post-extraction, bronchoscopic confirmation of complete removal and a follow-up CT scan are recommended to assess resolution and monitor for complications, such as residual bronchiectasis or scarring.

This study has several limitations that should be addressed when interpreting the findings. The limited sample size constrains statistical power and generalizability, as the clinical and radiological characteristics presented may not represent the full spectrum of chili pepper aspiration cases. The retrospective, single-center design introduces potential selection bias, since it includes only patients with persistent symptoms necessitating referral to a tertiary center, excluding milder cases that may resolve spontaneously or be managed at local hospitals. The follow-up duration was relatively short (6–12 months), precluding assessment of long-term complications such as progressive bronchiectasis, recurrent inflammation, or functional impairment. There are no validated imaging criteria for diagnosing chili pepper aspiration, and the CT features reported (V-shaped or annular hyperdensity) may overlap with other organic or inorganic foreign bodies. Finally, due to the retrospective nature, we were unable to collect detailed dietary history data (e.g., frequency of chili pepper consumption) that could inform risk stratification.

## Conclusion

This case series illustrates some clinical and radiological features of chili pepper fragment aspiration, particularly in elderly patients with impaired airway reflexes. Clinically, presentations are often nonspecific, mimicking common respiratory conditions such as chronic pneumonia, asthma, or malignancy. Radiologically, hyperdense bronchial opacities with V-shaped, U-shaped, or annular morphologies should raise concern for an organic foreign body of chili pepper fragment, though these findings are non-pathognomonic and may be obscured by distal migration or significant inflammation. Bronchoscopy remains the standard for definitive diagnosis and treatment, with cryoextraction potentially offering a valuable approach to intact chili pepper fragment removal and minimizing residual fragments. Preventive education regarding safe eating practices may benefit elderly individuals with dysphagia or neurologic impairment. Larger multicenter studies are needed to refine imaging protocols, establish management algorithms, and evaluate long-term outcomes post-extraction.

## Data Availability

The original contributions presented in the study are included in the article/Supplementary material, further inquiries can be directed to the corresponding authors.
