# Supplementary material for: Chili pepper aspiration in elderly patients: a case series highlighting diagnostic challenges and the role of cryoextraction
Source: Front Med (Lausanne). 2026 Feb 18;13:1751021. doi: 10.3389/fmed.2026.1751021 (PMC12956646; doi:10.3389/fmed.2026.1751021)
Supplement: Supplementary file 1 [file Table_1.DOCX]

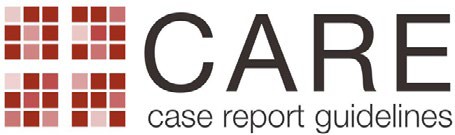

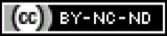

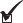
**CARE Checklist of information to include when writing a case report**

| **Topic** | **Item No** | **Checklist item description** | **Reported on Page Number/Line Number** |
| --- | --- | --- | --- |
| Title | 1 | The diagnosis or intervention of primary focus followed by the words “case report” | Title page |
| Key Words | 2 | 2 to 5 key words that identify diagnoses or interventions in this case report, including "case report" | p.1 abstract keywords |
| Abstract  (Structured summary) | 3a | Background: state what is known and unknown; why the case report is unique and what it adds to existing literature. | p.1 abstract ¶1 |
|  | 3b | Case Description: describe the patient’s demographic details, main symptoms, history, important clinical findings, the main diagnosis, interventions, outcomes and follow-ups. | p.1 abstract ¶2 |
|  | 3c | Conclusions: summarize the main take-away lesson, clinical impact and potential implications. | p.1 abstract ¶3 |
| Introduction | 4 | One or two paragraphs summarizing why this case is unique (may include references) | p.2 Introduction (entire section) |
| Patient Information | 5a | De-identified patient specific information | p.3 Case 1: 60-year-old man; p.4 Case 2: 73-year-old man |
|  | 5b | Primary concerns and symptoms of the patient | Case 1: 8-month non-productive paroxysmal cough; Case 2: 4-month right lower chest pain + 1-week productive cough |
|  | 5c | Medical, family, and psycho-social history including relevant genetic information | p.3–4: no relevant past history reported; no family or genetic history mentioned |
|  | 5d | Relevant past interventions with outcomes | Case 1: outside hospital moxifloxacin 14 days → modest transient improvement; forceps biopsy → chronic inflammation |
| Clinical Findings | 6 | Describe significant physical examination (PE) and important clinical findings | p.3 Case 1: ↓ breath sounds RLL, no adventitious sounds; p.4 Case 2: ↓ breath sounds RLL, no adventitious sounds |
| Timeline | 7 | Historical and current information from this episode of care organized as a timeline | p.3 Case 1 timeline: 0 mo initial CT → 8 mo repeat CT → transfer → bronchoscopy → 1 mo CT → 5 mo phone F/U; p.4 Case 2: 4 mo pain → 1 wk cough → CT → bronchoscopy → 3 mo phone F/U |
| Diagnostic Assessment | 8a | Diagnostic testing (such as PE, laboratory testing, imaging, surveys). | p.3–4: serial chest CT (lung & mediastinal windows), flexible bronchoscopy, forceps biopsy, cryoextraction |
|  | 8b | Diagnostic challenges (such as access to testing, financial, or cultural) | p.2 Discussion ¶1: absence of choking history, radiological subtlety, distal migration, inflammatory masking |
|  | 8c | Diagnosis (including other diagnoses considered) | p.3: initial diagnoses considered—obstructive pneumonia, neoplasm; final—chili pepper aspiration |
|  | 8d | Prognosis (such as staging in oncology) where applicable | p.5: residual bronchiectasis noted on 1-mo CT; long-term prognosis not fully assessed (short F/U) |
| Therapeutic Intervention | 9a | Types of therapeutic intervention (such as pharmacologic, surgical, preventive, self-care) | p.3–4: bronchoscopic cryoextraction; saline instillation; granulation tissue debridement |
|  | 9b | Administration of therapeutic intervention (such as dosage, strength, duration) | p.3: cryoprobe inserted into fragment, single freeze-adherence cycle; no drug dosages applicable |
|  | 9c | Changes in therapeutic intervention (with rationale) | None (cryoextraction successful on first attempt; no changes) |

| Follow-up and Outcomes | 10a | Clinician and patient-assessed outcomes (if available) | p.3: “patient satisfied… no special complaints”; p.4: “patient expressed satisfaction… no specific complaints” |
| --- | --- | --- | --- |
|  | 10b | Important follow-up diagnostic and other test results | p.3: 1-mo CT—residual RLL bronchiectasis with patchy opacities |
|  | 10c | Intervention adherence and tolerability (How was this assessed?) | Not formally assessed; no adverse events reported  None reported |
|  | 10d | Adverse and unanticipated events | None reported |
| Discussion | 11a | A scientific discussion of the strengths AND limitations associated with this case report | p.5 Discussion ¶ “This study has several limitations…” |
|  | 11b | Discussion of the relevant medical literature with references | p.2–5 Discussion (cites 22 references) |
|  | 11c | The scientific rationale for any conclusions (including assessment of possible causes) | p.5 Conclusion paragraph |
|  | 11d | The primary “take-away” lessons of this case report (without references) in a one paragraph conclusion | p.5 Conclusion: hyperdense CT opacities + cryoextraction efficacy + need for awareness in elderly |
| Patient Perspective | 12 | The patient should share their perspective in one to two paragraphs on the treatment(s) they received | p.3: “patient satisfied… no special complaints”; p.4: “patient expressed satisfaction… no specific complaints” |
| Informed Consent | 13 | Did the patient give informed consent? Please provide if requested | **Yes** |

Please leave this space alone as it will be supplemented by the editorial office when needed.
